# Fat Hounsfield Unit Reference Interval Derived through an Indirect Method

**DOI:** 10.3390/diagnostics13111913

**Published:** 2023-05-30

**Authors:** Marian Pop, Marius Mărușteri

**Affiliations:** 1 ME1 Department, George Emil Palade University of Medicine, Pharmacy, Science, and Technology of Targu Mures, 540139 Tirgu Mures, Romania; 2 Radiology and Medical Imaging Department, Tirgu Mures Emergency Institute for Cardiovascular Diseases and Heart Transplant, 540136 Tirgu Mures, Romania; 3 M2 Department, George Emil Palade University of Medicine, Pharmacy, Science, and Technology of Targu Mures, 540139 Tirgu Mures, Romania

**Keywords:** computed tomography, fat Hounsfield unit, reference interval, indirect method

## Abstract

Background: In vivo Hounsfield Unit (HU) values have traditionally been determined using direct CT image measurements. These measurements are dependent on the window/level used to examine the CT image and the individual conducting the fat tissue tracing. Methods: Using an indirect method, a new reference interval (RI) is proposed. A total of 4000 samples of fat tissues were collected from routine abdominal CT examinations. A linear regression equation was then calculated using the linear part of the cumulative frequency plot of their average values. Results: The regression function for total abdominal fat was determined to be y = 35.376*x − 123.48, and a 95% confidence RI of −123 to −89 was computed. A significant difference of 3.82 was observed between the average fat HU values of visceral and subcutaneous areas. Conclusions: Using statistical methods and the in vivo measurements of patient data, a series of RIs were determined for fat HU that is consistent with theoretical values.

## 1. Introduction

Measuring body fat is important in the evaluation of fatty lesions, but it is also pivotal in the evaluation of obesity. With more than 1.39 billion overweight adults [1] and more than 600 million obese individuals [1], there is a worldwide drive for research on obesity diagnostics and its health consequences (cardiovascular diseases, diabetes, and even cancers) [1].

Computed tomography (CT), along with magnetic resonance imaging (MRI), represent cross-sectional, noninvasive methods used for the measurement of abdominal fat distribution (with regard to visceral fat and subcutaneous fat components) and its correlation with various diseases and laboratory values [2].

CT measures the attenuation of X-ray beams passing through sections of the body from hundreds of different angles, and then, from the evidence of these measurements, a computer can reconstruct pictures of the body’s interior [3].

The acquired CT slice consists of a matrix of up to 1024 by 1024 volume elements (voxels), with each voxel being representative of the intensity of the transmitted radiation measured by detectors. From the intensity readings, the density or attenuation values of the tissues can be calculated, and an image is constructed as a corresponding matrix of picture elements (pixels) [4].

Each pixel is assigned a numerical value (CT number), which is the average of the attenuation values contained within the corresponding voxel [4]. This number is compared to the attenuation value of water and displayed on a scale of arbitrary units named Hounsfield units (HU) after Sir Godfrey Hounsfield. This scale assigns water an attenuation value (HU) of zero. Each number represents a shade of grey, with +1000 being assigned to white and –1000 being assigned to black [4].

Since each tissue has its own absorption coefficient (NIST [5]), establishing a reference interval (RI) for HU values is important both for establishing the fatty nature of the tissue and for measuring the abdominal fat compartments; a change in RI margins produces a change in fat tissue measurement dimensions.

Ceriotti defined the clinical laboratory RI as one that “when applied correctly to the population serviced by the laboratory, includes most of the subjects with characteristics similar to the reference group and excludes the others” [6]. A good RI will produce reproducible results.

Previous studies [7,8,9] have used direct measurements for determining the RIs, and while most RIs refer to the central 95% of the population of subjects, no RI can be completely “right” or “wrong”. 

The aim of this study was to establish reference intervals for Hounsfield units (HU) of abdominal fat in CT scans using a statistical approach: the indirect Hoffmann method.

## 2. Methods

In the following study, we used a different statistical approach for establishing fat HU intervals using the method described by Katayev et al. [10] in applying a visual computerized indirect Hoffmann method.

### 2.1. Participants

A retrospective study (20075/2013) was conducted using data collected from 50 routine consecutive CT examinations performed for abdominal clinical indications in the Tirgu Mures Emergency County Hospital in 2014 and 2015. 

The CT machine used to examine the patients was a Siemens Somatom AS+ 128, with spiral acquisitions and 5 mm slices with 30 f kernel reconstructions being captured.

To select a slice with enough abdominal fat and to ensure repeatability for a sample of four, slices were selected from the CT examination stack at selected anatomy landmarks:

Upper abdomen—through the left adrenal gland
Upper abdomen—through superior mesenteric artery ostium
Lower abdomen—through the umbilicus
Lower abdomen—through the anterior superior iliac spine

In case of multiple slices through the same anatomical landmark, the most cranial slice clear of artifacts was chosen.

After creating the slice database, the slices were opened in ImageJ 1.48 v. From each slice, 10 samples of subcutaneous fat and 10 samples of visceral fat were taken with an oval selection tool, and the average values of the HU values were recorded.

Some samples had a small area of “contamination” with nonfat tissue, and, to eliminate its impact on the data, the samples with a maximum HU higher than 0 were removed (nonfat tissue); the same was applied to those with <500 HU (samples including intestinal air).

### 2.2. Statistical Analysis

As previously described [10], the Chauvenet criterion for outliers was used multiple times for the detection and elimination of outliers. With Chauvenet criteria, a measurement (result) is eliminated if the probability of its occurrence is less than 1/(2 N), where N is the number of measurements in the data pool and is greater than 4.

After the elimination of outliers, the cumulative frequency for the average HU pixel value was calculated. The frequency of an HU value was taken as the number of times it appeared in the dataset divided by the total number of samples times 100.

$$F_{HU_{i}}=\frac{Count\;\left(HU_{i}\right)}{Count\;\left(total\;number\;of\;samples\right)\;}\times 100$$

The cumulative frequency of an HU value was: $CF_{HU_{i}}=\overset{i}{\underset{j=2}{\sum }}F_{HU_{i}}$, ordered by *HU_i_*

As per the indirect Hoffman method, the values that were plotted as a cumulative percentage and the straight-line component represent normally distributed data; they were used to interpolate a central percentage for reference intervals.

**Figure 1:**
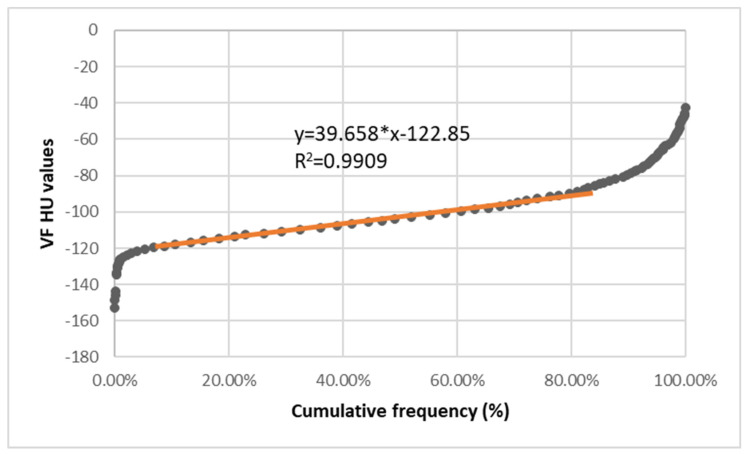
Visceral fat graph, with a linear portion of the graph being described by y = 39.658*x − 122.85 (R^2^ = 0.9909).

The visual analysis provides an effective approximation of the linear data, and once adequate linearity was obtained, the portion of the data pool that was linear was selected and used to determine the associated regression line (Figure 1).

Afterward, the best fit linear regression equation (y = αx + β) was determined using least squares analysis (where α = the slope and β = the intercept of the line).

The RI was determined from the linear regression equation using extrapolation of the preceding curve. 

The RI was calculated for 95% confidence (using x = 2.5% and 97.5%), where

Lower RI margin = α*2.5 + β
Upper RI margin = α*97.5 + β.

All statistical comparisons were conducted with a two-tailed T-test, assuming that the data were homoscedastic and had a significance level of 0.05.

## 3. Results and Discussion

A total of 3926 samples were analyzed after removing the outliers. The visceral fat samples were more numerous (1977 vs. 1949) and with a significant average value lower than the subcutaneous fat (−100.65 vs. −104.47, *p* < 0.05). 

**Figure 2:**
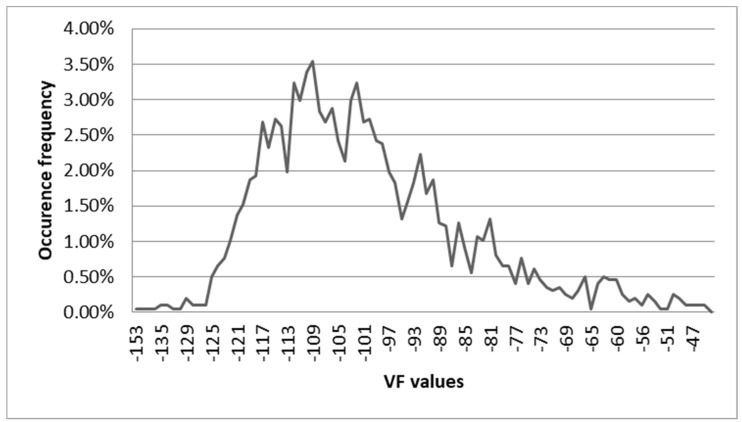
Graph of The Visceral Fat Hounsfield Units. Linear range −118: −88 HU. N = 1977 values (without outliers). Regression function y = 39.65*x − 122.85. Reference interval: −121.86: −84.18.

The occurrence frequency of fat HU values and their distribution showed similar characteristics, with a dataset skewed to the right (Figure 2) and with reference intervals ranging from −123 to 93 for subcutaneous fat and −122 to −84 for visceral fat (Table 1).

The regression functions for each type of abdominal fat were computed with a high coefficient of determination (>0.99), and they appear in Table 1.

Using the indirect Hoffmann method for indirect determination of the HU reference range, we obtained intervals that overlap most of the previously determined RIs. 

Since the first reported measurements of what will be called “Hounsfield units,” the values of the fat were determined to be negative, with fat having an absorption coefficient of only 10% of the water [11]; later measurements by Hounsfield extended the fat tissue values into a range from −90 to −70 UH [3]. All further determined reference intervals overlap the theoretical values and extend them, with our RI being the most restrictive. We consider our narrow interval to be due to our measurements being performed on “pure-fat” tissues, with the exclusion of fat-containing connective tissue.

Using the NIST (National Institute of Science and Technology) X-ray Mass Attenuation Coefficients for adipose tissue [5], dry air, and liquid water, and the HU formula, the fat HU values at 100 keV are −114 and for 150 keV are of −33. There is a small difference between these theoretical values and our experimental ones; nevertheless, it should be remembered that diagnostic CT uses 120 keV, and the adipose tissue in the organism will be found in different proportions in various structures. 

### 3.1. Previous Studies

Previous studies have investigated the use of fat Hounsfield Units (HU) to distinguish between different types of adipose tissue on CT scans, with advancements in CT technology and newer software applications allowing for better visualization of adipose tissue, leading to improved understanding of the relationship between fat HU and metabolic disease.

In order to segment the fat tissue, various thresholds are considered (Table 2), including (−140:−40), (−190:−30), (−250:−50), (−250:−20), or (−150:−50) [7,8,9,12], with suggested close agreements between different window widths for examination; however, the exact thresholds used can vary depending on the visual assessment of the radiologist and can be influenced by the CT detector technology and imaging processing software used.

While we considered using the average +/−3 standard deviations for measuring the reference interval, we consider that since the average HU values are not normally distributed, this could lead to inaccurate results. However, our results would have varied from those of Jensen [17], where the RI limits were determined by subtracting three SDs from the mean and adding two SDs to the average HU values.

Although the results suggest nearby RI for various types of abdominal fat, we found a 5% difference between the average abdominal and visceral fat values, with the subcutaneous fat being less radio-dense than the visceral fat. We consider this to be due to the structural differences and adipose tissue composition. No data were available to compare those findings.

The observed differences in HU values of visceral fat compared to subcutaneous fat indicate its denser and metabolically active nature. This difference has significant clinical ramifications because visceral fat is closely associated with metabolic diseases such as cardiovascular disease, diabetes, and certain cancers. Assessing the distribution and characteristics of visceral fat using HU values can provide valuable insight into a person’s health risks and aid in the development of preventative and diagnostic strategies.

This research has implications for diagnostic imaging in the context of obesity and metabolic disorders. The indirect Hoffmann method increases the precision and comprehension of Hounsfield units (HU) values for abdominal fat. This technique facilitates the identification, classification, and monitoring of conditions such as cardiovascular diseases, diabetes, and certain cancers. The precise determination of HU reference intervals increases the clinical utility of software applications that rely on accurate fat HU values. Overall, this research contributes to the development of more reliable imaging techniques and the improvement of the diagnosis and treatment of diseases associated with obesity.

Future research could expand the sample size by collaborating with multiple medical centers or radiology laboratories to collect data from a more diverse population to address this study’s limitations. A greater understanding of abdominal fat Hounsfield units (HU) across populations could be obtained by focusing on specific demographic groups, such as various age groups, genders, and ethnic backgrounds. Incorporating a longitudinal study design to evaluate changes in fat HU values over time, including the impact of treatments and lifestyle interventions, would also contribute to a more complete understanding of abdominal fat composition.

### 3.2. Limitations

It is important to acknowledge some limitations of our study. The first is the relatively small sample size which may restrict its generalizability to broader populations. Additionally, this study may have limitations in its ability to evaluate patients with low abdominal fat, particularly those in the pediatric or emaciated groups. Furthermore, variations in acquisition protocols may have led to minor differences in the obtained results. Moreover, as a retrospective, cross-sectional study, we were unable to assess the changes in fat HU values due to treatments or sports exercises, but we hope that future studies will address these questions in greater detail and provide a more comprehensive understanding of the factors that contribute to obesity.

Multiple acquisition parameters and machine types could be added to the database of HU values to minimize the limitations. By incorporating data from a variety of acquisition parameters and machine types, it is possible to gain a more comprehensive understanding of the range and variability of HU values across various imaging settings. Then, statistical analyses can be used to identify and mitigate the impact of these factors, ensuring that the determined reference intervals are robust and applicable to a variety of imaging scenarios.

## 4. Conclusions

Our method provided an abdominal fat tissue HU reference interval using the Hoffmann indirect method. This method was able to derive RIs for abdominal fat components, producing a result that was in strong agreement with scientific observations. This method has the potential to be easily implemented in a variety of radiology laboratories or hospitals, providing a valuable diagnostic tool for understanding obesity and metabolic diseases. 

The visceral fat RI was determined as (−121.86:−84.18 HU), and the subcutaneous fat RI was determined as (−122.98:−93.21 HU), with a statistically significant difference between them.

Although all fat HU values could be regarded as accurate, their goal should be clinical, with a solid reference interval enhancing the clinical value of software that relies on it.

## Figures and Tables

**Table 1:** Regression function and calculated reference intervals for abdominal fat determined via the computerization of the Hoffmann method for the indirect estimation of reference intervals.

	Equation	R^2^	RI
Total Abdominal Fat	y = 35.37*x − 123.48	>0.99	−122.59:−88.98
Subcutaneous Fat	y = 31.28*x − 123.71	>0.99	−122.98:−93.21
Visceral Fat	y = 39.65*x − 122.85	>0.99	−121.86:−84.18

**Table 2:** Comparison of reference intervals calculated via the Hoffmann method with RIs as reported and used in the past 3 years as reflected in the literature. Total abdominal fat RIs calculated: −123:−89 HU.

Previous Reports	Reported/Used RI	Absolute Difference (Upper/Lower)
Yu, 2023 [13]	−150:−50	27:−39
Yi, 2022 [14]	−195:−45	72:−44
Brian, 2022 [15]	−205:−51	82:−38
Maurovich, 2007 [16]	−195:−45	72:−44
Jensen, 2001 [17]	−149+/−12:−68+/−7	26:−21
Kvist, 1998 [7]; also used by Pellegrini, 2022 [18]; Baek, 2022 [19]; Barbalho, 2022 [20]; Jung, 2021 [21]; Lee, 2021 [22]	−190:−30	−67:59
Enzi, 1986 [8]	−250:−50	−127:39
Hounsfield, 1979 [3]	−90:−70	33:19:00
Hounsfield, 1973 [11]	−100 (−10% of water)	23:−11

## Data Availability

Not applicable.
